# Impact of Arieş River Contaminants on Algae and Plants

**DOI:** 10.3390/toxics11100817

**Published:** 2023-09-28

**Authors:** Adela Halmagyi, Anca Butiuc-Keul, Martin Keul, Cristina Dobrotă, László Fodorpataki, Adela Pintea, Aurel Mocan, Valeria Pop, Ana Coste

**Affiliations:** 1 Department of Experimental Biology, National Institute of Research and Development for Biological Sciences, Branch Institute of Biological Research Cluj-Napoca, 48 Republicii Street, 400015 Cluj-Napoca, Romania; adela.halmagyi@icbcluj.ro (A.H.);; 2 Faculty of Biology and Geology, Babeș-Bolyai University, 1 M. Kogălniceanu Street, 400084 Cluj-Napoca, Romania; 3 Centre for Systems Biology, Biodiversity and Bioresource, Babeș-Bolyai University, 5-7 Clinicilor Street, 400006 Cluj-Napoca, Romania; 4 Department of Horticulture, Sapientia University, 2 Sighișoarei Rd., 540485 Târgu Mureș, Romania; 5 Department of Chemistry and Biochemistry, University of Agricultural Sciences and Veterinary Medicine Cluj-Napoca, Calea Mănăstur 3-5, 400372 Cluj-Napoca, Romania; 6 Institute of Public Health Prof. Dr. I. Moldovan, 6 Louis Pasteur Street, 400349 Cluj-Napoca, Romania; 7 Doctoral School “Environmental Science”, Babeș-Bolyai University, 1 M. Kogălniceanu Street, 400084 Cluj-Napoca, Romania; 8 Faculty of Environmental Science and Engineering, Research Institute for Sustainability and Disaster Management Based on High Performance Computing, Babeș-Bolyai University, 30 Fantanele Street, 400294 Cluj-Napoca, Romania

**Keywords:** cytotoxicity, ecotoxicology, contaminants, water quality

## Abstract

The Arieş River (Western Romania) represents one of the most important affluents of the Mureş River, with great significance in the Mureş Tisza basin. The environmental quality of the Arieş basin is significantly affected by both historic mining activities and contemporary impacts. Thus, an evaluation of the effects of the main contaminants found in water (organochlorine pesticides—OCPs, monocyclic aromatic hydrocarbons—MAHs, polycyclic aromatic hydrocarbons—PAHs, and metals) on cyanobacteria and plants was performed. Among OCPs, hexachlorocyclohexane isomers, dichlorodiphenyltrichloroethane, and derivatives were detected in plants while admissible concentrations were detected in water. Among MAHs, high levels of benzene were detected both in water and in plants. The levels of PAHs exceeded the allowable values in all samples. Increased concentrations of metals in water were found only at Baia de Arieş, but in plants, all metal concentrations were high. The pH, nitrates, nitrites, and phosphates, as well as metals, pesticides, and aromatic hydrocarbons, influenced the physiological characteristics of algae, test plants, and aquatic plants exposed to various compounds dissolved in water. Considering that the Arieş River basin is the site of intense past mining activities, these data provide information about the impact on water quality as a consequence of pollution events.

## 1. Introduction

Because life cannot exist on Earth without water, it is crucial to preserve water supplies and the environment from all types of pollution. The availability and quality of water are two of the most important concerns that humanity has recently confronted [1]. Future water availability is uncertain due to rising freshwater demand brought on by population growth, altered land use, and climate change [2]. Water regulations were developed in the European Union (EU) Water Framework Directive of 2000 and were implemented in EU Member States in 2003. The new deadline of 2027 has replaced the initial 2015 deadline for achieving an “excellent status” [3]. This implies achieving an ‘excellent ecological and chemical state’ for surface waters and a ‘good chemical and quantitative status’ for groundwater. It also states that the water bodies’ status shall not degrade [3]. Achieving the 2030 Agenda for Sustainable Development is critical for future generations as the Sustainable Development Goals aim for universal access to and sustainable management of water and sanitation [1]. It is estimated that 80% of all industrial and municipal wastewater is discharged into the environment untreated, causing harm to human health and ecosystems [1]. In many parts of the world, wasteful use of water for food production has resulted from a lack of water valuation [4]. Pollution of water resources as a result of human activities (mining, metallurgical processing of metals, agriculture, domestic waste, etc.) entails multiple risks to human and animal health [5], ecological risks [6], and environmental risks [7]. Parasitic, viral, and bacterial infections [8]; gastrointestinal ailments [9]; cancer [10]; and neurological problems [11] are all dangers connected with polluted water. As a result, safeguarding water quality is critical to avoid these negative consequences. At the same time, people must be educated about the environment and the harmful impacts of water contamination.

The Arieş River is situated in Western Romania and represents one of the most important right-side affluents of the Mureş River. Due to its geographic position and its significance in the Mureş Tisza basin, the Arieş River and its water quality play a significant role in the preservation of our ecosystems [12]. The Arieş River basin contains both inactive and active metal mines that may contribute to existing and future pollution. Two of the most important mining centers situated in the Arieş River catchment are Roşia Poieni and Roşia Montană [13,14]. In Romania, water quality is monitored according to the principles of the Integrated Water Monitoring System (IWMS), restructured according to European Directives. The Law 310/2004 amending the Law 107/1996 includes the provisions of the Framework Directive 60/2000/EEC and other EU Water Directives. In summary, there are 12 classes of quality indicators that are used to monitor water quality: oxygen regime, water aggression indicators, salinity indicators, presence of nutrients, general inorganic contaminants, general organic contaminants, microporous heavy metals, microporous pesticides, radioactivity, microbiology, biology, and flow. The allowable values for basic indicators of surface water pollution are shown in NTPA 001/2002 [15]. Some persistent contaminants have long-term effects on aquatic environments even after the pollution source is removed. The effects are diverse, including biological consequences (disappearance of plant and animal species, changes in aquatic biocenosis, development of thermophilic algae and anaerobic microorganisms, eutrophication, compromising agriculture via irrigation with polluted water), economic consequences (increased cost for water treatment, providing water from other sources, cost for treating people who become ill due to water pollution), and social consequences (compromising recreation areas, compromising urban and tourist areas, affecting protected areas, population migration due to the deterioration of water quality).

Numerous studies over the years have examined the Arieş River’s water quality based on the evaluation of physico-chemical parameters [16,17], contamination with heavy metals [12,18,19], with pesticides and organochlorines [20], chemical measurements of pore water and sediment in tailings [13], the composition of tailings’ minerals [21], the measurement of dissolved anions and cations by using capillary electrophoresis [22], or the effect of contaminated water on germination and growth rates of wheat seeds [23]. As previously shown, the index of saprobity of water exhibits a growing tendency from upstream to downstream in the Arieș River. Thus, the water quality upstream of Câmpeni is considered oligosaprobic (class I–II), being clear or very slightly polluted. Downstream of Câmpeni, the water belongs to class II, β-mesosaprobic or moderately polluted. The river water downstream of Baia de Arieș could be included in class II–III, i.e., β-α-mesosaprobic level with moderate to strong pollution, but in Sălciua—upstream and downstream—it could be included in class III–IV, i.e., β-α- and α-mesosaprobic level, being strongly polluted [24].

Considering that the huge quantities of tailings and waste generated over the years by mining still represent a permanent pollution source for surface and underground waters in the area, it is important that the water quality of the Arieş River should be monitored regularly. Moreover, the impact evaluation of water quality on organisms living in water ecosystems is also important, providing valuable information about the effects of long-term pollution and the cumulative effects of different chemical compounds dissolved in the water body. As many of previous articles focused on monitoring the pollution of the Arieș River with metals resulting from mining activities, the objective of our study was the investigation of the physiological and cytological effects of the main contaminants found in water such as organochlorine pesticides (OCPs), monocyclic aromatic hydrocarbons (MAHs), and polycyclic aromatic hydrocarbons (PAHs), usually used as solvents and trace and heavy metals on algae and plants, giving information about the water quality and its impact on the environment, organisms, and human health. All these compounds are highly persistent and bioaccumulating contaminants.

## 2. Materials and Methods

### 2.1. Sampling and Physico-Chemical Evaluation of Water

Water and aquatic plant samples were collected in September 2007 from various locations along the Arieş River (NW Romania): (1) Câmpeni upstream, (2) Câmpeni downstream, (3) Baia de Arieş upstream, (4) Baia de Arieş downstream, (5) Sălciua upstream, (6) Sălciua downstream, (7) Turda upstream, (8) Turda downstream, (9), Câmpia Turzii upstream, (10), Câmpia Turzii downstream, and (11) Abrud inflow. The collection points were selected based on the impacts of industrial activity (in Turda and Câmpia Turzii) and past mining activities (in Roşia Montană, Câmpeni, Abrud, and Baia de Arieş) on water resources.

The following physico-chemical parameters have been determined for water samples: pH and concentrations of NO_3_ (mg L^−1^), NO_2_ (mg L^−1^), PO_4_ (mg L^−1^), Cu (μg L^−1^), and Fe (μg L^−1^).

### 2.2. Assessment of Pollutant Compounds

The presence of pollutant compounds was determined in water and aquatic plant samples collected from the sites mentioned above within 24 h after collection. All the experiments carried out with algae and plants were set up within 24 h after the collection of water samples.

#### 2.2.1. Analysis of Organochlorine Pesticides—OCPs

Gas chromatography (GC) with electron capture detection (ECD) and mass spectrometry (MS) were used to determine the following organochlorine pesticides (OCPs): hexachlorocyclohexane isomers (α, β, γ and δ-HCH), dichlorodiphenyl trichloro ethane (op’DDT, pp’DDT) and its metabolites (op’DDE, pp’DDE), aldrin, dieldrin, and endosulfan sulfate. The SR EN ISO 6468:2000 international reference standard method was used [25]. The samples were processed and analyzed following these steps: (i) Liquid–liquid extraction of organochlorine pesticides from water and solid–liquid extraction from plants, using high-purity petroleum ether or n-hexane as the extraction solvent—in case of plant material, the samples were crushed, then homogenized with the extraction solvent, and the extraction was performed via ultrasonication of the homogenate. The extract was separated by using centrifugation; (ii) anhydrous sodium sulfate dehydration of the organic extract; (iii) concentration of the extract using a rotary evaporator; and (iv) gas chromatographic analysis of the concentrated extract. This step was performed using an HP-608 capillary separation column (length 30 m, inner diameter 0.35 mm) and an electron capture detector. Separation was achieved via linear temperature programming of the column. Component identification is based on retention time, and quantitative analysis was performed using the external standard method and quantitative standard solutions (LGC Standards, Germany). The method used has a detection limit of 0.0001 μg/L of water.

#### 2.2.2. Monocyclic and Polycyclic Aromatic Hydrocarbons

The monocyclic aromatic hydrocarbons (MAHs) were determined using the following protocol: (1) water sample processing via “head space”, which entails equilibration at 90 °C and (2) liquid–solid extraction in the case of plants (after pre-shredding of samples) using n-hexane. Ultrasounds were used to extract the sample, which was then concentrated in a rotary evaporator. Gas chromatography was used to analyze the gaseous phase in equilibrium with the thermally balanced sample. The components were separated on a capillary column, HP-5 (15 m × 0.53 mm × 0.25 m), using linear programming of the column temperature. A flame ionization detector was used. The qualitative analysis was based on retention time. The concentration was calculated using standard solutions of benzene, toluene, xylene, and ethylbenzene. Chemically pure water contaminated with known quantities of compounds was used as a control. The limits of detection (LODs) were 0.002 mg/L for water and 0.002 mg/kg for plant material. Water and plant samples were measured in triplicate, and the results are presented here as mean values.

The polycyclic aromatic hydrocarbon (PAH) measurements were accomplished using a gas chromatography–mass spectrometry detector (Agilent, Santa Clara, CA, USA). The extraction method involved the following stages: (1) liquid–liquid extraction of polycyclic aromatic hydrocarbons using the mixture of n-hexane-methylene chloride (3:2) as extractant and (2) liquid–solid extraction using ultrasounds for plant material. Extraction with n-hexane-methylene chloride was performed after preliminary grinding and homogenization of the samples, concentration of the extract in the rotary evaporator, and gas-chromatographic analysis of the concentrated extract. The separation was performed on a BP-5 capillary column (50 m × 0.32 mm × 0.25 m). Flame ionization detection combined with mass spectrometry was applied. The standard solutions included naphthalene (Nap), acenaphthylene (Acy), fluorene (Flo), phenanthrene (Phe)+anthracene (Ant), pyrene (Pyr), benzo(b)fluoranthene B(b)F, benzo(k)fluoranthene B(k)F, benzo(a)pyrene B(a)P, and benz(a)anthracene B(a)A+chrysene (Chr). The limits of detection (LODs) were 0.002 mg/L for water and 0.002 mg/kg for plant material. Water and plant samples were measured in triplicate, and the results are presented here as mean values.

#### 2.2.3. Trace and Heavy Metals

Water samples were treated with concentrated nitric acid to adjust the pH to 1–2. Plant samples were dried at 105 °C until they reached a constant weight. The utilized method is based on the measurement of metal concentrations in acidulated samples in an air–air flame or through electrothermal atomization. For the determination of metals in water or plant material, the samples were previously mineralized. Control and water samples were treated with concentrated mineral acids, then oxygenated water, and heated to evaporation temperature. Following evaporation, the process was repeated with the same amount of nitric acid. Control and plant samples were treated with concentrated nitric acid and perchloric acid and heated on a sand bath to the evaporation temperature. The above mentioned steps were repeated until the residue turned white and was completely dry. Then, the samples were treated with 4% nitric acid and brought to a final volume (50 mL) through successive washings. The concentrations of lead (Pb), cadmium (Cd), copper (Cu), chromium (Cr), nickel (Ni), zinc (Zn), manganese (Mn), and iron (Fe) were determined using atomic absorption spectrometry in the air–acetylene flame (Ni, Zn, Fe) and via electrothermal atomization (Pb, Cd, Cu, Cr, Mn) using a spectrophotometer (Analyst 700, PerkinElmer, Woodbridge, ON, Canada) [26]. The absorbances were related to the calibration curve, and the corresponding metal concentrations were determined. The metal concentrations determined in water samples were expressed in mg/L and in plant samples in mg/kg dry weight. Standard solutions for each metal were prepared via sequential dilution of stock standards to 1000 ppm for the calibration curve.

### 2.3. Assessment of Water Quality via Ecotoxicological Studies with Algae

The standard ISO 8692:2012 [27] was used to analyze the water toxicity of the Arieş River. The tests were performed using water samples from the following sampling sites: Baia de Arieş downstream, Sălciua downstream, Turda upstream, Câmpia Turzii downstream, and Abrud inflow.

The test species was *Scenedesmus opoliensis* (PG. Richter) (Chlorophyceae, Chlorococcales), a fast-growing coenobia with four cells. This standard protocol describes a method for determining the suppression of algal development by soluble chemicals and mixtures in water. Four stock solutions were produced, autoclaved, and kept in the dark at 4 °C. These stock solutions were diluted to achieve the final nutrient concentrations in the test solutions. Exponentially growing cultures of selected green algae were exposed to various concentrations of the polluted water under defined conditions. The initial cell density in the test cultures was approximately 10^4^ cells per mL. The test solutions were incubated for 72 h, with cell density measurements taken at 24 h intervals. The inhibition of growth was determined in relation to a control culture. Four water concentrations (25%, 50%, 75%, and 100%) were prepared by adding suitable aliquots to algal precultures. All operations were carried out under sterile conditions to avoid contamination. After inoculation, the test flasks were incubated in a culture chamber at 23 ± 2 °C under continuous illumination (400–700 nm spectral range and a light intensity of 60–100 μmol m^−2^ s^−1^). Cell density measurements were made, using the direct counting method, of living cells under a microscope with counting chambers. The pH was measured at the beginning of the test and after 72 h (Table 1). The test design included two replicates for each test concentration. The cell density in each flask was determined at 24, 48, and 72 h after the test started. The mean value of cell density for each concentration and for the control was plotted against time (0–72 h).

The percentage inhibition of the specific growth rate at each concentration (Iμt) was calculated according to the following formula:

Iμt = (μc − μt)/μc × 100

where μc is the mean control-specific growth rate and μt is the mean specific growth rate for the test concentration t. The acute endpoint, ErC50, concerning the concentration of tested water that results in a 50% reduction in growth rate relative to control within 72 h, was determined.

### 2.4. Evaluation of the Cytotoxic Potential of Water from Arieș

The Allium test [28] was used to assess the genotoxicity of water from Arieş River. Onion bulbs (*Allium cepa* L., cv. Galbena de Stuttgart) (2n = 16) approximately 3–4 cm in diameter were placed on the necks of 200 mL Erlenmeyer flasks filled with tap water for rhizogenesis. When the roots reached 1 cm in length, the bulbs were transferred on top of other flasks filled with water collected from the sampling sites described previously. Tap water was used as control.

The mitotic activity in the root meristem was assessed 5 days after the bulb transfer in water from Arieș. Root apices (2 cm in length) were fixed in Carnoy solution (2 h) and then washed in 70% ethanol to remove any traces of acetic acid and stored in Eppendorf tubes in 70% ethanol at 4 °C until examination. Root tips were treated with 1% acetic anhydride and macerated in 1 N HCl (10 min) at room temperature. Staining was made with 1% aceto-orcein (prepared in 45% acetic acid) for 24 h at room temperature. For each water sample, including the control, at least three root tips were microscopically examined in four repetitions.

The mitotic index was calculated by analyzing (1000 cells per water sample) the ratio between the cells in division (n) and the total number of analyzed cells (N) according to the following formula:

MI (%) = n/N

The relative frequency of division phases (I) was determined by calculating the prophase (p), metaphase (m), anaphase (a), and telophase (t) index as the ratio between the number of cells in each phase (d) and the total number of cells in division (D) according to the following formula:

I _p. m. a. t_ (%) = d _p. m. a. t_/D

The frequency of abnormal mitosis, in particular the appearance of micronuclei, anaphase and telophase bridges, chromosomal fragments, and binuclear cells, was calculated as a percentage of the total number of cells in division.

### 2.5. Analysis of Carotenoids, Chlorophylls, and Fatty Acids from Phospholipids and Glycolipids

Total carotenoids were extracted from fresh aquatic plant material (2 g) with a mixture of methanol/ethyl acetate/petroleum ether (1:1:1, v/v/v). The obtained extract was repeatedly washed in a separation funnel to remove water and ethanol. The organic phase (acetate/petroleum ether) was dried over anhydrous sodium sulfate and evaporated to dryness using a rotatory evaporator (35 °C). The dry residue was dissolved in acetone (10 mL) and added to Ambersep 900 OH resin (1 g) in order to remove chlorophyll [29]. The samples were centrifuged, and an aliquot was used for spectrophotometric determination of total carotenoid content (Jasco V-530 UV-VIS spectrophotometer, Jasco Europe SRL, Cremella, Italy) according to the formula proposed by Britton et al. [30].

Another aliquot was evaporated, re-dissolved in the mobile phase, and analyzed on an HPLC system consisting of LC20 AT Shimadzu pumps, a Waters 990 photodiode detector, and a YMC C30 column (25 cm × 4.6 mm, particle diameter 5 μm). The mobile phase consisted of two solvent mixtures: A—methanol: tert-butyl-methyl-ether/water (81:15:4)— and B—consisting of the same components in a ratio of 6:90:4. A linear gradient from 1% B to 100% B in 90 min was used, with a flow rate of 0.8 mL/min and an injection volume of 20 μL. Individual carotenoids were identified by comparing their retention times, elution order on the C30 column, and UV-VIS spectra (λmax, spectral fine structure %III/II) with those of the available standards (β-carotene, lutein, and zeaxanthin) and with literature data for the other carotenoids. The composition of carotenoids extracts was expressed as the area percentage of each identified compound from the total area of carotenoids in the samples. Carotenoids standards were (Sigma-Aldrich, Saint Louis, MI, USA) used.

For chlorophylls analysis, the samples (0.2 g) were homogenized and repeatedly extracted with 80% acetone in water until the residue was colorless. The samples were filtered, and the chlorophyll concentration was determined according to the following formulas:

chl a (mg/g wet biomass) = (12.7 × E663 − 2.6 × E645) v/g

chl b (mg/g wet biomass) = (22.9 × E645 − 4.6 × E663) v/g

Here,

chl a—chlorophyll a
chl b-—chlorophyll b
E645—sample extinction at 645 nm
E663—sample extinction at 663 nm
v—volume of acetone extract
g—weight of samples extracted with acetone

Total lipids were extracted using the Folch method [31]. The phospholipids and glycolipids were separated using thin-layer chromatography on silica gel plates using, as the mobile phase, a mixture of methyl acetate:1-propanol:chloroform:methanol:KCl 0.25% (25:25:28:10:7.5) [31]. The standards used for identification of glycolipids and phospholipids were as follows: monogalactosyldiacylglycerol (MGDG), digalactosyldiacylglycerol (DGDG), phosphatidylethanolamine (PE), phosphatidylglycerol (PG), phosphatidic acid (PA), phosphatidylinositol (PI), phosphatidylserine (PS), and phosphatidylcholine (PC) (Sigma-Aldrich). The spots of glycolipids (MGDG and DGDG) and phospholipids (PC, PA, PI, PS, PG, and PE) were scraped together from the plate to form the two polar lipid fractions. Lipid samples on silica gel were transesterified as methyl esters (FAME) with 14% BF3/methanol and analyzed using gas chromatography with FID detection. A Shimadzu GC 17A system with a flame ionization detector (FID) and a Crompack Silica 25 MXO capillary column (25 m × 0.25 mm i.d., film thickness 0.25 mm) were used. The temperatures were as follows: 5 min at 150 °C, 4 °C/min gradient to 235 °C, and 5 min at 235 °C. The temperature of the injector was 260 °C, and that of the detector was 260 °C. The carrier gas used was helium. The identification of the fatty acid methyl esters was based on the comparison of their retention times with those of the FAME standards. The methyl esters of palmitic acid (16:0), palmitoleic acid (16:1), stearic acid (18:0), oleic acid (18:1 (9c), linoleic acid (18:2), and linolenic acid (18:3) were the standards used for the GC analysis (Sigma-Aldrich).

### 2.6. Chlorophyll Fluorescence Measurements

Young bean (*Phaseolus vulgaris* L.) plants were used to test the influence of polluted water on photosynthetic light use efficiency. Following germination, control plantlets were watered with 10 mL of dechlorinated tap water per plant every third day. A total of 60 plantlets (each experimental variant with five replicates) in pots filled with vermiculite were used. Before sowing, the substrate was saturated with Hoagland’s mineral nutrient solution to provide all the essential inorganic macro- and micronutrients. The plants were grown for three weeks in a growth chamber (Sanyo MLR-351H) under a 16 h photoperiod (photon flux density of 530 µmol m^−2^ s^−1^) at 23 °C during the light periods and 20 °C in the dark phases and 60% relative air humidity. The position of every plant in the growth chamber was changed randomly once every three days.

Parameters of induced chlorophyll *a* fluorescence were measured, in situ and in vivo on the first pair of leaves that developed after the greening of cotyledons, on five plantlets for each experimental variant with an FMS2 chlorophyll fluorometer (Hansatech, King’s Lynn, UK). The measurements were repeated twice on the two sides of the main vein and on the adaxial surface of leaf blades. To determine the conventional (non-modulated) parameters of chlorophyll fluorescence, the plantlets were dark-adapted for 15 min prior to measurements in order to stop every previous photosynthetic process in the leaves [32]. Ground chlorophyll fluorescence (Fo) was measured by exposing the leaves to a very weak red light flash (0.1 µmol m^−2^ s^−1^) while the transitory maximal fluorescence (Fm) was generated by a strong, saturating light pulse (10,000 µmol m^−2^ s^−1^ for 0.5 s). The potential quantum yield of photosystem II (PSII) was determined using both the Fv/Fm and the Fv/Fo ratios (where Fv = Fm − Fo) [33]. Fo and Fm were related to the same chlorophyll *a* quantity. For this reason, after the fluorescence measurements were ended, 0.25 g fresh weight of the leaf blades used for fluorescence measurements was ground in a mortar in the presence of magnesium carbonate and the photosynthetic pigments were extracted with 25 mL of 80% (*v*/*v*) acetone. The extracts were centrifuged for 10 min at 4000× *g* and 4 °C, and the absorbance of the supernatants was measured at 663 nm [34]. The pulse amplification-modulated chlorophyll fluorescence parameters were registered in constantly illuminated leaves upon application of modulating light flashes. Steady-state fluorescence was measured under continuous illumination with 800 µmol m^−2^ s^−1^ actinic light, and then, a saturating white light flash was used to record the modulated maximal fluorescence Fm’. The values of Fs and Fm’ were used to establish the effective quantum yield of PSII (Φ = (Fm’ − Fs)/Fs [35]. The non-photochemical quenching of the fluorescence signal (NPQ) was expressed with the relation (Fm − Fm’)/Fm’ [36]. The vitality index of the photosynthetic apparatus, determined at saturating irradiance (white light with 3000 µmol m^−2^ s^−1^ for 5 min), was expressed as the fluorescence decrease ratio or relative fluorescence decrease (Rfd) with the relation (Fm − Fs)/Fs [37]. The chlorophyll fluorescence data were statistically evaluated with the R statistical package, using the Shapiro–Wilk test for normality analysis and Bartlett’s test for establishing the homogeneity of variances. Data sets were represented as means ± standard deviation (SD, n = 5). Differences were considered significant at *p* < 0.05, as determined with a one-way ANOVA followed by a Tukey HDS test.

## 3. Results

### 3.1. Assessment of Physico-Chemical Parameters of Water

The main physico-chemical parameters such as pH, anions, and cations assessed in water samples are shown in Table 1. The pH values were similar and near-neutral at most of the sampling sites except Turda upstream, where the pH was 6.85. The pH increased during algal growth (after 72 h). PO_4_ anions were detected at all sampling sites, but NO_3_ and NO_2_ were detected only at some of the sampling sites. Fe cations were detected at all sampling sites, and Cu as well, except at the Turda upstream sampling site.

**Table 1:** Physico-chemical (NO_3_, NO_2_, PO_4_ expressed in mg/L, and Cu, Fe in μg/L) * parameters of Arieş River water.

	Initial pH	pH after 72 h	NO_3_	NO_2_	PO_4_	Cu	Fe
Baia de Arieș downstream	7.31 ± 0.11	8.15 ± 0.14	0.8 ± 0.06	0.42 ± 0.05	0.89 ± 0.08	0.01 ± 0.01	48 ±2.6
Sălciua downstream	7.36 ± 0.12	8.12 ± 0.13	0	0.065 ± 0.04	0.89 ± 0.07	0.12 ± 0.05	58 ± 4.2
Turda upstream	6.85 ± 0.13	7.15 ± 0.11	0	0	0.66 ± 0.05	0	440 ± 10.2
Câmpia Turzii downstream	7.85 ± 0.16	8.61 ± 0.14	0.8 ± 0.05	0.42 ± 0.03	0.89 ± 0.06	0.01 ± 0.01	48 ± 3.5
Abrud inflow	6.4 ± 0.15	6.52 ± 0.15	0	0	0.89 ± 0.04	0.05 ± 0.02	440 ± 12.3

* Data represent the mean values ± standard deviation (SD) of three replicates. The pH after 72 h represents the pH changes during the algal growth.

### 3.2. Assessment of Pollutant Compounds

#### 3.2.1. Organochlorine Pesticides

The concentrations of OCPs varied between 0.001 and 0.041 μg/L in water samples and 0.49 and 50.33 μg/kg DW in aquatic plants (Table 2). Two hexachlorocyclohexane isomers (α-HCH and γ-HCH) were mainly identified in water samples from upstream areas, while β- and δ- HCH isomers were mainly present, along with the other isomers, in water samples from the downstream sites Câmpia Turzii and Turda and Abrud flow. Among HCH isomers, the highest concentration in water samples was registered for α-HCH (0.041 μg/L). The pesticide endosulfan sulfate was identified only in six water samples (0.006–0.016 μg/L) while the 1,1-dichloro-2,2-bis(4-chlorophenyl)ethane—DDE and dichlorodiphenyltrichloroethane (DDT) isomers were present only in one water sample from Câmpia Turzii downstream in relatively low concentrations (0.003–0.006 μg/L). OCPs were detected in much higher concentrations in aquatic plants (Table 2) compared to the analyzed water samples. OCP concentrations in plant samples ranged from 0.49 to 50.33 μg/kg DW (dry weight) for HCH isomers and 0.2 to 4.63 μg/kg DW for dichlorodiphenyl tri-chloro ethane (DDT) and its metabolites (DDEs). Aldrin and dieldrin were absent in both water and plant samples, while high levels (0.49–1.80 μg/kg DW) of endosulfan sulfate were detected in plant samples from Sălciua, Câmpia Turzii, and Turda localities. Overall, the range of identified pesticides was much wider in plants than in water samples, with almost all samples containing not only HCH isomers but also DDE and DDT isomers. The highest OPC concentrations were registered for γ-HCH (50.33 μg/kg) in aquatic plant samples from Sălciua downstream.

#### 3.2.2. Monocyclic and Polycyclic Aromatic Hydrocarbons

The levels of MAHs and PAHs detected in the Arieş River basin are presented in Table 3 and Table 4. Among the assessed MAHs, benzene was the main pollutant in water samples (0.07–8.01 mg/L), while in plants, the highest levels (6.98 mg/L) were registered in samples from Turda downstream. Ethylbenzene was not detected in any of the analyzed samples, whereas xylene was present in low concentrations in water samples (0.28–0.51 mg/L) and in the highest concentration (3.02 mg/kg DW) in plant samples from Turda downstream. Toluene was detected mainly in water samples (0.19–0.48 mg/L) at all sites and only at three sites in plant material, with the highest concentration (0.76 mg/kg) being at Câmpeni upstream (Table 3).

Naphthalene and acenaphthylene were not found in any plant or water samples from any sampling site, while pyrene and benzo(a)pyrene were only found in plant samples from Câmpeni upstream and downstream, respectively (Table 4). The other studied PAHs were present in different concentrations, either in water samples or in plant samples, only at some sites. Most of the PAHs were identified at all sampling sites and were mainly present in plant material and less in water. The highest concentration of PAHs was 53.0 mg/kg of benzo(b)fluoranthene and benzo(k)fluoranthene in plant material from Sălciua downstream. Benzo(a)pyrene (BaP, 0.81 mg/kg) was found only in plant material from Câmpeni upstream.

#### 3.2.3. Trace and Heavy Metals

Some of the metals investigated were not detected in Arieş water, such as Pb, Cr, and Ni. Cadmium was detected only in Abrud inflow (0.008 mg/kg), with the concentration being above the imperative value according to EU Directive 75/440/EEC [38]. Copper was also detected, but only at Sălciua upstream, Baia de Arieş upstream, and Abrud inflow, where the concentrations were above the imperative value according to EU Directive 75/440/EEC. Manganese was detected at all sampling sites, with concentrations ranging between 0.034 mg/kg in Câmpia Turzii downstream and 5.136 mg/kg in Abrud inflow. Zinc and iron were detected at all sampling sites as well. Zn concentrations at all sampling sites were below the target or imperative values according to 75/440/EEC. Iron was detected in lower concentrations, ranging between 0.071 mg/kg at Câmpia Turzii upstream and 0.962 mg/kg at Câmpeni downstream and Sălciua upstream. The highest Fe concentration was detected in water collected from Sălciua downstream (5.462 mg/kg) (Table 5).

In plants collected from different sampling sites, all trace metals were detected, with the highest concentrations being in plants from the downstream sites of the cities, except in plants collected from Sălciua upstream, which showed higher concentrations of all metals than plants from Sălciua downstream (Table 5). Higher concentrations of Pb were detected in plants collected from the sector Sălciua–Câmpia Turzii–Turda–Baia de Arieş. Cadmium concentrations were similar in plants collected from all sampling sites, with the highest concentration being found in plants from Baia de Arieş downstream (0.134 mg/kg). High concentrations of copper were found in plants from Sălciua upstream (8.037 mg/kg), Baia de Arieş upstream (10.559 mg/kg), and Baia de Arieş downstream (25.509 mg/kg). Manganese concentrations were variable in plants from different sample sites, with the lowest concentration being found in plants from Sălciua downstream (2.657 mg/kg) and the highest concentration being found in plants from Baia de Arieş downstream (89.38 mg/kg), but high Mn concentrations were found in plants from Sălciua upstream (34.034 mg/kg) and Baia de Arieş upstream (38.84 mg/kg). Chromium concentrations were similar in plants from all sampling sites except the plants from Câmpia Turzii upstream and downstream (0.36 mg/kg). The highest concentrations of nickel were detected in plants from the Câmpia Turzii upstream and downstream sampling sites (about 3.2 mg/kg), Turda upstream and downstream (about 3.3 mg/kg), and Baia de Arieş downstream (3.057 mg/kg). Zinc concentrations were higher in plants from Sălciua upstream and Baia de Arieş upstream (about 6.4 mg/kg), with the highest concentration being detected in plants from Baia de Arieş downstream (18.585 mg/kg). Lead was detected in high concentrations in plants from all sampling sites, with the highest values found at Câmpia Turzii upstream and downstream (about 380 mg/kg).

### 3.3. Assessment of Water Quality via Ecotoxicological Studies with Algae

The main physico-chemical parameters that affect algal growth are pH, type of medium, preculture conditions, and strain selection. The pH increased during algal growth, shifting to a more alkaline reaction due to the depletion of dissolved carbon dioxide, which is used in algal photosynthesis (Table 1). A strong negative correlation was established between the growth medium concentration and the growth rate of the algal cultures (Table 6). The highest values were recorded for Câmpia Turzii, downstream from the industrial zone (r = −0.91), and the lowest values were recorded for the sampling site located downstream of Baia de Arieş (r = −0.69), a former gold mine that is currently closed. The percentage inhibition of algal culture ranged between 11.91% and 82.40% according to the tested concentrations (Table 7). Considering the effect of chemical parameters of polluted water on growth inhibition, significant positive correlations with pH (r = 0.61), nitrate (r = 0.58), and nitrite (r = 0.56) were recorded when the algal culture in the study was grown in river water (100%). For the concentrations of 25%, 50%, and 75%, very weak or no significant correlations with these parameters were found. The ErC50 values were between 22.05 for the Baia de Arieş downstream sampling site and 60.7 for Turda upstream, indicating a lower ecotoxic effect upstream of Turda compared to downstream of Baia de Arieş, where the concentration of test water, which resulted in a 50% reduction in growth rate relative to the control, was much lower.

### 3.4. Evaluation of the Cytotoxic Potential of Water from the Arieș River

The water samples from different sites exhibited varying impacts on the mitotic index, frequency of mitotic divisions, and occurrence of chromosomal aberrations in onion-root meristematic cells. The chromosomal aberrations showed the highest value (2.5) in plants treated with water from Abrud inflow, while the lowest value was noted alongside control in plants treated with water from Sălciua downstream and Câmpia Turzii upstream (0.4) (Table 8). Concerning instances of abnormal mitosis, these were present in cells treated with water from Sălciua upstream (0.1), Turda upstream (0.1), Câmpia Turzii downstream (0.1), and Abrud inflow (0.2) (Table 8).

### 3.5. Analysis of Carotenoids, Chlorophylls, and Fatty Acids from Phospholipids and Glycolipids

**Figure 1:**
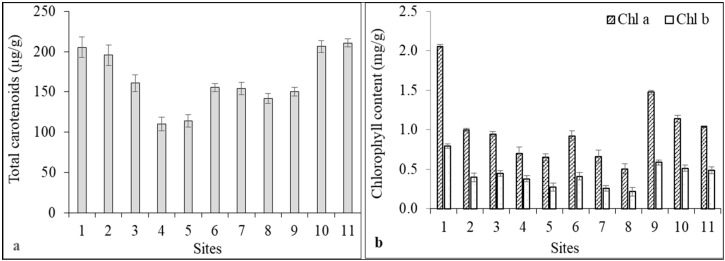
Pigment content in aquatic plants collected from the course of the Arieş River: (**a**) total carotenoids; (**b**) Chlorophyll a and Chlorophyll b. The 11 samples represent the collection sites: (1) Câmpeni upstream, (2) Câmpeni downstream, (3) Baia de Arieş upstream, (4) Baia de Arieş downstream, (5) Sălciua upstream, (6) Sălciua downstream, (7) Turda upstream, (8) Turda downstream, (9) Câmpia Turzii upstream, (10) Câmpia Turzii downstream, and (11) Abrud inflow. The data represent the mean values ± SD of three replicates.

In the present study, we measured chlorophyll a (Chl a), chlorophyll b (Chl b), and total carotenoids from aquatic plants using classic spectrophotometric methods. Moreover, individual carotenoid content was determined using HPLC-PDA. The total carotenoid content ranged between 110.23 and 210.80 µg/g, with the highest concentration being for Abrud inflow and Câmpeni upstream and the lowest for Sălciua downstream and Câmpia Turzii upstream (Figure 1a). Chl a ranged between 0.51 and 2.06 mg/g while Chl b content was 0.21–0.79 mg/g in aquatic plant samples (Figure 1b). The Chl a/Chl b ratio ranged between 1.86 and 2.59. The lowest values for chlorophyll concentration were recorded for the samples from Turda downstream, Câmpia Turzii upstream, Sălciua downstream, and Turda upstream.

Photosynthetic tissues primarily contain the same major carotenoid pigments, which include beta-carotene, lutein, violaxanthin, and neoxanthin. Several other minor carotenoids can be identified in certain species: antheraxanthin, zeaxanthin, β-cryptoxanthin, 5,6-lutein-epoxide, α-carotene and lactucaxanthin [26]. The HPLC-PDA analysis of the aquatic plant samples highlighted the same pattern, as shown in Table 9. Lutein was the major pigment in all the samples, representing 46–63% of the total carotenoids, followed by β-carotene, with 27–41%; neoxanthin; and violaxanthin. Zeaxanthin and antheraxanthin were found in low amounts in all analyzed samples, below 1% and up to 3.2%, respectively. Differences in the composition of carotenoid pigments existed, but these variances cannot be solely attributed to environmental factors since the collected samples encompassed various species.

In the present study, we extracted and separated two major classes of polar (complex) lipids, glycerophospholipids and glyceroglycolipids, and determined their fatty acid profiles using gas chromatography (Table 10 and Table 11). The major fatty acids in aquatic plant phospholipids were palmitic acid (26–42%) and linolenic acid (22–35%), followed by linoleic acid (13.6–21%). The variations between the fatty acid concentrations of the samples were quite important and could have been caused both by external factors and by the variability of the plant material. Nevertheless, it was observed that the decrease in linoleic acid coincided with an increase in the proportion of linolenic acid and saturated acids. The Double Bond Index (DBI), as a measure of unsaturation degree, varied from 1.95 to 3.53 in the case of phospholipids, with an average value of 2.92.

Concerning glyceroglycolipids, a much higher percentage was observed for linolenic acid (43–65%) than otherwise expected. The linoleic acid content was lower in most of the glyceroglycolipid samples, as were the contents of palmitic and stearic acids. In general, the degree of unsaturation of glycolipids was much higher, with a percentage of 63–75% unsaturated fatty acids, compared to phospholipids with 45–59% AGN. This profile is even better depicted by the DBI values, which ranged from 4.69 to 8.91 with an average of 5.82.

### 3.6. Chlorophyll Fluorescence Parameters

In bean leaves grown under similar photon flux densities and temperature regimes, Fo values significantly decreased in plants treated with water from Baia de Arieş downstream, Câmpia Turzii downstream, and Abrud inflow (Table 12). Chemical analyses of the water revealed that in water from Baia de Arieş downstream, there are higher concentrations of certain metals (especially copper, manganese, and iron) and monocyclic aromatic hydrocarbons (mainly benzene and toluene). At Câmpia Turzii downstream, the water contained organochlorinated pesticides and higher amounts of xylene while in Abrud inflow, there were higher quantities of both metals (mainly zinc and manganese) as well as monocyclic and polycyclic aromatic hydrocarbons (especially fluorene).

**Figure 2:**
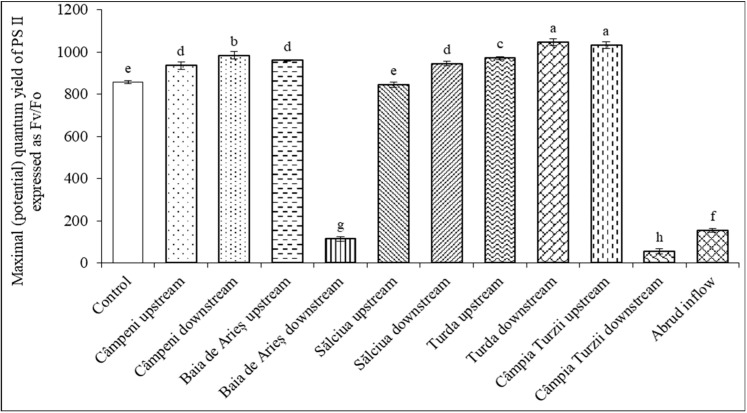
Influence of water samples from the Arieş River on the maximal quantum yield of photosystem II (PSII), expressed as the Fv/Fo chlorophyll fluorescence ratio, in leaves of bean plants. Vertical bars represent ± SD from means (n = 5); different letters indicate significant differences at the *p* < 0.05 level.

In bean plants treated with water from the Arieș River, a pronounced decrease in Fm (expressed on a chlorophyll unit basis) could be registered under the influence of the same water sample, which also caused a decay in ground fluorescence, i.e., Baia de Arieș downstream, Câmpia Turzii downstream, and Abrud inflow (Table 12). One of the most largely used parameters of induced chlorophyll fluorescence in plant-stress physiological studies is the potential or maximal quantum yield of PSII (expressed as Fv/Fm or as Fv/Fo). The most significant declines of the Fv/Fm ratio were identified in plants treated with water from Câmpia Turzii downstream and Baia de Arieș downstream (Figure 2).

**Figure 3:**
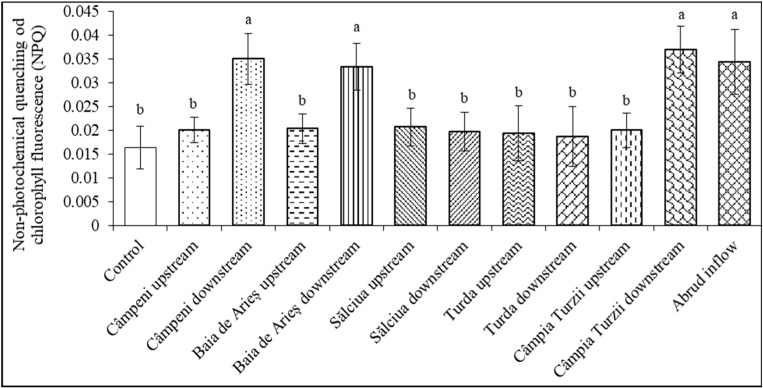
Influence of water samples from the Arieş River on the non-photochemical quenching of chlorophyll fluorescence in leaves of bean plants. Vertical bars represent ± SD from means (n = 5), different letters indicate significant differences at the *p* < 0.05 level.

In bean plants treated with water from the Arieș River, significant decreases in the effective quantum efficiency were recorded in two cases: under the influence of organic contaminants (organochlorinated pesticides and monocyclic aromatic hydrocarbons) from Câmpia Turzii downstream and in the presence of water polluted with trace metals from the mining region of Baia de Arieș (Table 12). In leaves, a statistically significant increase in NPQ values was induced by water from Câmpeni downstream (where polycyclic aromatic hydrocarbons and higher concentrations of ferric ions were detected), Baia de Arieş downstream (with high concentrations of metals and monocyclic aromatic hydrocarbons), Câmpia Turzii downstream (with organochlorinated pesticides), and Abrud inflow (with trace metals and certain aromatic hydrocarbons) (Figure 3).

**Figure 4:**
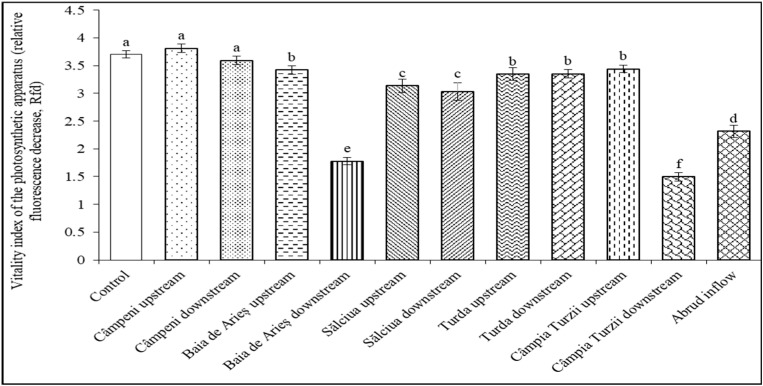
Influence of water samples from the Arieş River on the vitality index of the photosynthetic apparatus, expressed as relative fluorescence decrease levels, in leaves of bean plants. Vertical bars represent ±SE from mean values (n = 5); different letters indicate significant differences at the *p* < 0.05 level.

The chlorophyll fluorescence decrease ratio (Rfd), also known as the vitality index of the photosynthetic apparatus, is frequently used as an overall vitality marker in stress physiology studies. The influence of polluted water on the photosynthetic performance of bean leaves showed that the vitality index of the photosynthetic apparatus (expressed as Rfd) was a sensitive marker for the presence of reduced concentrations of inorganic and organic water contaminants (mainly trace metals, pesticides, and aromatic hydrocarbons). The vitality index values were not significantly modified in the case of water from Câmpeni upstream and Câmpeni downstream, suggesting that in this region, the water does not exert an obvious polluting effect on the examined plants. A moderate but statistically significant decrease in the vitality index was registered under the influence of water from Baia de Arieş upstream, Turda upstream and downstream, and Câmpia Turzii upstream. A further decrement could be observed in plants treated with water from Sălciua upstream and downstream. The lowest values of the vitality index were obtained for plants treated with water from Baia de Arieş downstream, Câmpia Turzii downstream, and Abrud inflow (Figure 4).

## 4. Discussion

### 4.1. Analysis of Organochlorine Pesticides

OCPs are the most widely encountered contaminants, being described as persistent and bioaccumulating substances prone to long-range transportation [40]. Although severely restricted or banned in many countries, OCPs still appear as contaminants, both in food as well as in the environment [41]. In our study, the major contaminants were mainly found in aquatic plant samples and were represented by HCH isomers, DDTs, and derivatives, as well as endosulfan sulfate. Among HCHs, the dominant isomers were β-and γ-HCH, with these data being consistent with the intensive use of γ-HCH (lindane) as a pesticide over the years in the studied area and the challenge associated with the environmental degradation of HCHs [20].

According to the European Directive 2008/105/EC for surface waters, the maximum limit for hexachlorocyclohexane is 0.04 μg/L and for endosulfan, 0.01 μg/L [42]. The OPC levels in the water samples analyzed in our study were slightly higher than MACs for α-HCH (0.041 μg/L for α-HCH) and endosulfan sulfate (0.016 μg/L). OCPs were found to be mainly accumulated in aquatic plants (15.87 μg/kg for α-HCH; 50.33 μg/kg samples for γ-HCH; 3.78 μg/kg DW for DDTs; 4.63 μg/kg for DDEs; and 1.80 μg/kg for endosulfan sulfate). This might be explained by OCPs’ cumulative properties and the bioaccumulation potential of many living organisms including plants, and, thus, their potential migration along food chains [43].

We detected residual OCPs that have been extensively used in the past and have, due to their long-time persistence in the environment, accumulated in higher concentrations in aquatic plants. However, OCPs (0.1445–0.3164 μg/L) were detected in two surface water samples collected in 2015 from the Arieş River, with HCH isomers, mainly γ-HCH, being the most predominant [20]. The authors showed that there was a fresh input of lindane (γ-HCH), a pesticide originating from dump deposits of the former Turda Chemical Plant and supposedly used only in past agricultural practices [20]. However, it seems that many of these pesticides (DDT, technical HCH—prohibited in Romania since 2003, lindane—forbidden since 2007), are still illegally applied and are continuously detected in relatively high concentrations in the environment [20,44]. There are several other studies occasionally reporting OPC levels significantly above the regulatory limits and not only in the network of internal rivers [45,46].

### 4.2. Monocyclic and Polycyclic Aromatic Hydrocarbons

MAHs are composed of single-aromatic-ring compounds, such as benzene, toluene, ethylbenzene, and xylenes (BTEX), that are widely used as solvents [47]. Among the investigated MAHs, only benzene can be found on the watch list of substances for Union-wide monitoring in the field of water policy pursuant to Directive 2008/105/EC of the European Parliament and of the European Council [42]. According to this directive, the maximum allowable concentration (MAC) for benzene in surface waters is 50 μg/L. The levels registered for benzene in the present study in both water and plant samples were extremely high, ranging between 0.07–8.01 mg/L in water samples and 0.59–6.98 mg/kg DW in plant samples. In particular, water samples from Abrud inflow showed exceptionally high levels of benzene (8.01 mg/L). This could potentially be attributed solely to leaks originating from hazardous waste sites containing benzene or spills of petrol and petroleum products. Moreover, this collection point is located near the Roşia Poieni mine, the second largest copper deposit in Europe. Contamination of water, especially with benzene, is considered a major health issue since benzene is a highly volatile compound that is rapidly absorbed following inhalation [48]. Benzene is not persistent in surface waters, being either volatilized or degraded by bacteria, but if it exists deeper down in the groundwater, it is more persistent [49]. It has a half-life lasting from a few days to weeks, up to 28 days in groundwater while, in the air, it has a half-life of about 5 days [48,49]. The published research on water contamination with benzene is mainly related to regions hosting different industries: point emissions [48], gasworks [49] and chemical plants [50]. MAHs are often reported in water and wastewater [51] and are known as toxic chemicals [52]. BTEX concentrations reported in effluents, spills, and top soil were up to 1.5 mg/kg (mg/dm^3^ for liquid samples) [53].

The values registered in water samples for toluene and xylene were within the acceptable limits (1 and 10 mg/L, respectively) according to WHO guidelines for drinking water [54].

According to the EU’s Drinking Water Directive (EU) 2020/2184, the sum of benzo(b)fluoranthene, benzo(k)fluoranthene, benzo(ghi)perylene, and indeno(1,2,3-cd)pyrene must not exceed 0.1 μg/L in drinking water [55]. However, the maximum limit imposed by the European Directive 2008/105/EC for fluoranthene only in surface waters was 0.1 μg/L [42]. In addition, the amount of benzo(a)pyrene must not exceed 0.1 μg/ in surface waters, while there are no applicable limits for BbF and BkF [42]. In our study, PAH concentrations exceeded the allowable values in all samples and at all sites compared to drinking water limits. Thus, BbF and BkF were the main PAH contaminants registered in plants while BaA and Chr, and Phe and Ant, were the only PAHs detected in water samples at three sites: BaA and Chr at Abrud inflow (2 µg/L) and Câmpeni upstream (30.3 µg/L) and Phe and Ant only at Sălciua upstream (2.2 µg/L). According to published data, the presence of Chr indicates coal combustion, while BaA and BbF may originate mostly from vehicle emissions and the burning of fossil fuels [56]. The PAH levels detected in our study were higher than those registered in surface waters and sediments collected from Tisza and its tributaries, ranging from 1.22 to 260.26 ng/L in water samples and 4.94 to 10.62 μg/kg in sediment [57] or in the Olt River, with total PAH concentrations in water ranging from 1.3 to 46.2 ng/L and sediment content from 1.78 to 614.04 μg/kg [58]. PAHs are widely distributed in the environment [59,60]. The presence of the identified aromatic hydrocarbons can be due to the spillage of petroleum products or the discharge in the Arieş River of waste waters resulting from economic activities containing these compounds. It was underlined that generally, PAH pollution is closely associated to the level of socio-economic development in the local area, the resident population, and the industry [59]. PAH pollution has been concentrated mainly in water pollution in specific river segments or reservoirs [61]. The presence of benzene in surface waters represents a high risk because this compound is carcinogenic. Given its presence in gasoline, it appears as a pollutant whenever such fuels reach water, air, and soil.

### 4.3. Trace and Heavy Metals

Despite the long history of metal mining activity in Romania, there has not been a systematic evaluation of the effects of metal dumping into local rivers. Previous data showed that the Arieş River is polluted mostly with Cu and Zn (due to mining activities in the Arieş hydrographic basin), but also with significant levels of Cd and Fe [62]. The Arieş River is of particular interest because of the significant damages caused by precious metal exploitation. The metal concentrations found in our study in Arieş River water were below those detected in previous studies; this was probably due to the cessation of mining in the area. Thus, the metal concentrations declined with distance from the mine and time due to the self-decontamination processes in the zone [63]. Previous results showed exceeding-EU-imperative values for Cd, Cu, and Zn by 7.6, 58, and 1.8 times in the Abrud River downstream of the Bucium mine [64]. Cu and Zn concentrations increase downstream of the mining-affected tributary Arieş, contributing to the contamination of the sediments in the Arieş River, especially in Abrud and Baia de Arieş, as shown in our study. The main source of Cu in the water of the Arieş River is the Roşia Poieni, located 12 km upstream from Baia de Arieş [65]. A previous study showed that despite the significant metal inputs from the Roşia Montană and Roşia Poieni mining areas, the metal concentrations in the Arieş River were high only for Mn, Cu, Fe, and Pb [14]. This explains our results as well; lower concentrations were found most probably due to the dilution effect that occurs as a result of the low flow rate of the tributary Arieş compared with the flow rate of the Arieş River [66]. Excepting Baia de Arieş, where, usually, increased concentrations of Cr, Zn, As, Se, Cd, Pb, and U were found, at the other sites, only traces of heavy metals were found. Dilution is probably the main factor that explains the low metal concentrations in the Arieş River, but other processes, such as adsorption, absorption, chemical precipitation, and complexing, occur and lead to changes in the composition of the surface waters [22].

Generally, the surface water in the middle part of the Arieş River basin, near and downstream of the gold mine impoundment, showed high pollution with metals [19], but more distant sectors were less polluted, as shown in the present study. Other studies showed that mining activity in the Arieş basin has affected metal concentrations in surface water, such as those of Pb, Cu, Cd, and Zn. The high concentrations of these metals and the low pH values suggest that, even closed, the mines and tailings dams represent continuous pollution sources for natural waters [12]. In contrast to the results on Arieş water, tailings were found to be highly contaminated with various metals including As, Cu, Cd, and Pb, which could be considered potential sources of soil, surface water, and groundwater contamination [21]. Although the heavy metal concentration did not exceed the maximum allowable levels, except for Fe, Mn, and Pb, the Arieş River water was categorized as susceptible due to persistent long-term pollution [17]. Based on multiple pollution indices, the central section of the Arieş River, particularly downstream from the gold mine impoundment, exhibited a notably elevated pollution level. Potential Ecological Risk Index values for soil and sediments were in the order Cu > Ni > Pb > Hg > Cr > As > Mn > Zn > Cd and As > Cu > Cr > Cd > Pb > Ni > Hg > Mn > Zn, respectively [19].

However, heavy metals accumulated in the sediments had had an adverse impact on the aquatic flora. All metal concentrations in plants were high, especially those of Pb, Cu, and Zn in the sector Sălciua–Câmpia Turzii–Turda–Baia de Arieş. The metal concentrations in plant species irrigated with contaminated water were previously examined. The results varied significantly across plant species and sampling sites, exerting a substantial influence on the plants’ absorption capacity as previously discussed. Generally, the results showed that vegetables contained high concentrations of metals [67]. This explains the high concentration of metals found in plant samples from aqueous ecosystems that are continuously exposed to heavy metal pollution.

### 4.4. Assessment of Water Quality via Ecotoxicological Studies with Algae

In previous studies on *Scenedesmus obliquus* [68], the highest rate of biomass-specific growth and biomass productivity were associated with relatively low pH (6 pH) and relatively high temperatures (30 °C). The pH higher than 8 measured in this study may be one of the causes of algal growth inhibition. This aspect may be one of the shortcomings of the standard protocol because the depletion of carbon dioxide (in solution and in the headspace of the closed flasks) is an unusual situation in natural habitats. For optimal acidic growth in laboratory conditions, some compensatory measures, such as adapting the algal culture volume/headspace volume ratio, reducing pH, and increasing bicarbonate concentration, should be considered to maintain carbon dioxide availability. In water samples from Turda upstream, no nitrates, nitrites, or copper were detected, but a high level of iron was recorded, indicating a low level of pollution and oligotrophic conditions. Water samples from Câmpia Turzii downstream and Baia de Arieş contained nitrates, nitrites, and traces of copper. For these sites, the main sources of nitrate contamination are fertilizers and animal waste from agricultural areas adjacent to the water course. The level of phosphates was high and constant along the river. The usual sources of phosphate contamination are sewage, industrial waste, fertilizer, and runoff from agricultural sites. At high levels, phosphates sustain algal growth and eutrophication. The iron concentrations from Turda upstream and Abrud inflow were higher (440 μg/mL) compared to other sites due to local industrial-specific contamination.

Mining activity-induced pollution persists over an extended period, and waste containing heavy metals could continue to be released through rainfall. The highest differences among the growth rates compared to control were recorded for Turda upstream and Abrud inflow, where the highest level of iron was also registered.

It is considered that the growth rate is more appropriate against deviations in test conditions, allowing improved interpretations and comparisons between studies [69]. Even if the amounts of nitrates and nitrites are low, the cumulative effect of different factors, such as pH, may explain these results. Weak-to-medium negative correlations with copper (−0.49) and iron (−0.34) were recorded for the river water. The copper induced an increased trend of inhibition in relation to the concentration of the culture medium (r ranging from 0.47 at Iμ25% to 0.92 at Iμ75%). According to some perspectives [70], the currently accepted standard protocols employed within regulatory frameworks contain several shortcomings with regard to the physico-chemical and biological aspects of algal toxicity.

### 4.5. Evaluation of the Cytotoxic Potential of Water from the Arieș River

The *Allium* test was widely used to establish the level of cytotoxicity and genotoxicity in water [71,72]. Biological indicators have the advantage of sensitivity in detecting contaminants. The effects of tested waters on the mitotic index were quite moderate, varying insignificantly between control and the lowest value obtained for Baia de Arieş downstream. These results demonstrate the lack of phytotoxic effects or disturbances regarding cell multiplication in the *Allium* root meristem under treatment. On the other hand, the results regarding chromosomal aberrations showed a certain cytogenotoxic effect of water from certain locations. However, no micronuclei were observed, the occurrence of which has frequently been reported in cases of waters contaminated with heavy metals and cyanides [73]. Regarding the cytogenetic effect of waters (based on the *Allium* test) polluted with heavy metals and cyanides in Bulgaria, a decrease in cell division and the presence of abnormal mitoses were reported [73]. Although the evaluated waters did not exhibit significant cytotoxic effects in the *Allium* test, they still have deteriorated water quality and pose a threat to all organisms relying on them.

### 4.6. Analysis of Carotenoids, Chlorophylls, and Fatty Acids from Phospholipids and Glycolipids

It is worth mentioning that in the majority of the samples, there was a strong correlation between the chlorophyll content and the total carotenoid content. While plants from Câmpeni upstream and Abrud inflow presented the highest amounts of both chlorophylls and carotenoids, those collected from Sălciua downstream, Câmpia Turzii upstream, and Turda upstream exhibited the lowest pigment content. Moreover, plants from Baia de Arieş downstream had the lowest ratio of Chl a/Chl b. Plants from Câmpeni upstream, which showed the lowest level of contamination with trace and heavy metals, organochlorine pesticides, and PAHs, displayed the highest content of photosynthetic pigments. Plants from Sălciua upstream and Turda downstream had likewise accumulated significant levels of lead, iron, and chromium.

There are numerous studies showing that the quantitative and qualitative modifications of the membrane lipids play critical roles in inducing plant tolerance to stress, including xenobiotics like heavy metals, pesticides, etc. Key events among these modifications include the release of linolenic acid from membrane lipids by lipases and changes in unsaturation degree, which are mediated by desaturases [74]. PUFAs are released from membrane lipids and act as modulators of the signal transduction brought on by stress-inducing factors, particularly linoleic acid. Furthermore, it is well documented that xenobiotics, apart from their direct impact on fatty acid biosynthesis, can trigger the oxidation of highly sensitive polyunsaturated fatty acids. This oxidative process leads to changes in the composition of membrane fatty acids, finally compromising membrane activity [75,76].

Plants exhibit varying responses when subjected to the stress induced by heavy metals. Therefore, examining the fatty acid composition in two halophyte species (*Sesuvium portulacastrum* and *Mesembryanthemum crystallinum*) exposed to high doses of cadmium showed that in the case of *M. crystallinum*, the content of linolenic acid (C18:3) decreases and linoleic acid (C18:2) increases, while in *S. portulacastrum*, no significant changes in the composition were observed [77]. Differences may appear within the same species, depending on the organ analyzed. The decreases in the level of unsaturated fatty acids, fatty acid length (increase C16:0 to C18:0 ratio), and unsaturation degree (DBI) were also observed in the case of membrane lipids from the roots of cucumber seedlings treated with Cd, Ni, and, to a lesser extent, Cu [78]. The same effect was reported for spinach leaves in response to cadmium contamination [79]. However, in a more recent study, PUFA levels increased and oleic acid decreased in wheat plants treated with cadmium [80]. Although there have been fewer studies on the effect of heavy metals on lipid metabolism in aquatic plants, the mechanisms involved seem to be similar to those in terrestrial plants. Some authors have investigated the effect of copper ions (100 µM) on the lipid composition of subcellular membranes in *Hydrilla verticillate* [81]. In addition to the decrease in photosynthetic pigments, copper exposure induced lipid peroxidation, enhanced membrane permeability, decreased the lipid content, and affected the lipid and fatty acid composition of the membranes of chloroplasts, mitochondria, and microsomes. The profiles of fatty acids in total lipid extracts revealed that the main species were C18:3 (29–52%), C16:0 (18–27%), and C18:2 (11–18%), depending on the type of membrane. The C18:3/C16:0 ratio of chloroplast lipids decreased from 2.9 (control) to 0.7 in copper-treated cells after 24 h due to an increase in saturated fatty acids and a decrease in unsaturated fatty acids, mainly in the chloroplasts and microsomes.

In addition to membrane lipids (phospholipids, glycolipids, suberin, and cutins) that act as structural barriers to the environment, plants also contain neutral lipids (triacylglycerols), which are energy substrates in various metabolic processes. Polar lipids (like glycerophospholipids, glyceroglycolipids, sphingholipids, and cholesterol) are the major constituents of plant membranes. Hence, plants are characterized by a specific lipid and fatty acid composition within each cellular compartment and organelles. While PG, DPG, and PI can be found in thylacoids, phosphatidylcholine (PC) and phosphatidyl-ethanolamine (PE) are the most significant phospholipids and are the major constituents of extrachloroplastic membranes (the endoplasmic reticulum, the plasma membrane, the membranes of the Golgi apparatus, peroxisomes, glyoxysomes, the nucleus, and mitochondria). Thylakoids are distinguished by their so-called lateral asymmetry and have a completely distinct lipid composition from the other membranes, consisting predominantly of glyceroglycolipids. MGDG and PG are mainly found in the outer membrane and DGDG and SQDG in the inner one. Generally, phospholipids have two major fatty acids, which exist in the ratios 18:2 and 16:0. Both the ratio between phospholipids and their composition in fatty acids are closely dependent on the membrane type and on the plant material. Due to fluctuations in the levels of unsaturated fatty acids, which are recognized for their impact on membranes’ physical properties, plants respond to biotic and abiotic stress by modifying the fluidity of their membranes [74].

### 4.7. Chlorophyll Fluorescence Parameters

Biotic and abiotic stresses highly influence photosynthesis in plants; therefore, studying photosynthesis represents an effective way to reveal the mechanisms of their adaptation to different environments [82]. Chlorophyll fluorescence (Fo) is considered a useful tool to investigate the action mechanisms of adverse environmental factors, being an early and sensitive indicator of abiotic stress and a reliable marker of the protective capacity of plants against photooxidative damage and other mechanisms of stress tolerance [32,33].

Measurements of Fo reflect that water pollution with heavy metals and organic xenobiotics may affect light energy capture and transfer in the photosynthetic pigment antennae of leaves, impairing the accumulation of a suitable quantity of energy for the primary production of new plant metabolites. The fact that certain water samples (e.g., from Baia de Arieș downstream to Turda and Câmpia Turzii upstream) increased the value of Fo may be related to a compensatory reaction through which the plants, upon sensing a moderate stress condition due to mild water pollution, extended their light-harvesting antennae in order to avoid a decrement in light energy income. Decreased Fo values were also reported when different plant species were exposed to copper toxicity and oxidative stress conditions, while increments of Fo were recorded under the influence of heat shock and excessive light intensity [83]. Exposure to chromium also modified the value of this parameter in maize seedlings [84].

Our results suggest that the same water contaminants that disturb the organization of the light-harvesting complexes also impair the photochemical reactions on the acceptor side of PSII as revealed by the transitory maximal fluorescence (Fm) parameter. Similar decrements in Fm values were reported for plants exposed to prolonged heat stress, heavy metal toxicity, and herbicides that withdraw electrons from PSI [85,86]. The results concerning the Fv/Fo ratio suggest that these river sectors contain the highest quantities of contaminants (mainly organochlorinated pesticides at Câmpia Turzii and metals and monocyclic aromatic hydrocarbons at Baia de Arieş), which affect the capacity of plants to use light energy in photosynthesis.

It was demonstrated that under the influence of certain abiotic stress factors, especially when plants are exposed to mild stressors, the potential quantum yield of PSII, expressed as the Fv/Fo ratio instead of Fv/Fm, may be used as a more sensitive physiological marker of moderate disturbances to the photosynthetic apparatus [33]. This is also the case for water contaminants existing in the Arieș River, which had more amplified effects on the Fv/Fo ratio than on Fv/Fm. These modified values indicate that minor alterations in water quality induce protective reactions in the photosynthetic apparatus that aim to better cope with the adverse influences of polluting agents. The effective quantum efficiency of PSII (Φ) in plants exposed to ambient light provides a measure of the linear electron transport rate in thylakoid membranes; thus, it may be considered a functional indicator of overall photosynthesis [85].

An indicator of the protective capacity of plants against adverse environmental factors that impair photochemical reactions and enhance photo-oxidative threats is the non-photochemical quenching of chlorophyll fluorescence (NPQ). Under normal conditions, its numeric value is very small, but as stressors induce defensive reactions, its value increases significantly, reflecting that tolerance has been developed [34]. Similar increments of NPQ to those that we obtained in this study have been previously reported [84] when green microalgae were grown in water polluted with micromolar concentrations of chromium (6+), cadmium, and nickel. Furthermore, increased NPQ values were recorded when plants were exposed to various environmental stress conditions that inhibited the photochemical reactions of photosynthesis and induced the dissipation of excess energy as heat [35,36].

The chlorophyll fluorescence decrease ratio (Rfd) is directly correlated to the net carbon dioxide assimilation rate of leaves, thus being an indicator of the capacity of plants to increase their net biomass under given environmental conditions or contaminants [37,83]. A decrease in the concentration of chlorophyll and carotenoid pigments has been observed in aquatic plants (*Avicennia marina* and *Lemna trisulca*) exposed to cadmium, zinc, and copper ions [87,88]. More recently, a study showed that in the case of the aquatic macrophyte *Potamogeton pectinatus* L., a concentration of copper above 1 μM inhibited photosynthesis and decreased the levels of chlorophyll and carotenoids [89]. When *Ceratophyllum demersum* L. was exposed to Cd and Pb, the levels of chlorophyll a and b and carotenoids decreased with increasing metal concentration and time [90]. When other plant species were exposed to copper and chromium in similar conditions, they responded differently. For instance, *Eichhornia crassipes* accumulated chromium in the roots, and the metal treatment increased chlorophyll in the leaves, while *Salvinia biloba* and *Pistia stratiotes* were negatively affected at the level of photosynthetic pigments [4]. *Eichhornia* sp. and *Pistia* sp. also showed significant decreases in chlorophyll a and chlorophyll b with increases in the concentration of chromium [91].

Certain aquatic plants can act as hyperaccumulators able to remove heavy metals alongside with other contaminants; this is an important practical trait for implementing aquatic phytoremediation strategies. As an example, *Pontederia cordata* can tolerate low concentrations of cadmium and sequester it in the roots, but at high doses, chlorophylls and carotenoids are significantly reduced (by more than 60%) [92].

## 5. Conclusions

Our study revealed that the main contaminants in aquatic plants were organochlorine pesticides (OCPs) and derivatives, as well as trace and heavy metals. This implies that these dangerous compounds were still present and persistent in the environment. Both water and plant samples, notably in the Abrud inflow region, contained high concentrations of benzene. This suggests a possible source of pollution, maybe from petroleum-related activities or hazardous waste sites, which can have negative health implications. The permitted limits for the quality of drinking water were surpassed by polycyclic aromatic hydrocarbons (PAHs) in the water samples. These substances are indicative of pollution sources such industrial discharges or petroleum products, notably in the downstream area of Baia de Arieș. While trace and heavy metal concentrations in water samples were below previously reported levels, most probably due to mining activity cessation, high concentrations of metals like Pb, Cu, and Zn were found in plants in certain areas, suggesting ongoing pollution in the sector Sălciua–Câmpia Turzii–Turda–Baia de Arieş.

Water pollution also revealed cytogenetic effects, such as chromosomal aberrations, on plants, indicating potential genetic damage caused by the contaminants. However, micronuclei, more severe indicators of genotoxicity, were not observed. Moreover, parameters of induced chlorophyll fluorescence proved to be early and sensitive markers of the influence of trace metals and organic xenobiotics on the light-energy use efficiency of primary producers. This method could be introduced as a tool for the prevention or early detection of pollution in the routine analysis of water quality by water management authorities, being extended to different primary producers of aquatic ecosystems.

Implementing a long-term monitoring program for Arieș River water quality is crucial for assessing the environmental and public health risks associated with persistent contaminants and ensuring the protection of aquatic ecosystems. Consequently, the studies can be expanded to encompass a broader array of taxonomic groups, including microorganisms, invertebrates, fish, and birds. Although many studies have reported various mining and industrial operations as the main sources of water pollution in the area, further investigations are required to pinpoint the exact origins of the main contaminants identified in our study (benzene and PAH contamination, OCPs, trace and heavy metals) and to thoroughly evaluate the ecological repercussions on aquatic plant and animal species. Furthermore, research should be conducted to examine the potential of aquatic plants in bioremediation efforts to identify indicator animals and bacteria relevant for characterizing the quality of aquatic environments, thereby exploring sustainable strategies for pollution mitigation. Finally, it is crucial to assess the potential health risks to the local population resulting from exposure to polluted water and aquatic products in order to ensure their protection. The environmental and public health concerns connected with water pollution in the Arieș River can be better understood and managed by undertaking these further investigations and acting on the prescribed conclusions.

## Figures and Tables

**Table 2:** Organochlorine pesticide concentrations in water (μg/L) and plant (μg/kg DW)samples collected from the Arieş basin.

		α-HCH	β-HCH	γ-HCH	δ-HCH	Op-DDT	pp-DDT	op-DDE	pp-DDE	Aldrin	Dieldrin	Endosulfan Sulfate
Câmpeni upstream	water	0.002	ND	0.003	ND	ND	ND	ND	ND	ND	ND	ND
	plants	10.11	1.12	6.45	0.86	0.75	ND	2.88	1.56	ND	ND	ND
Câmpeni downstream	water	0.003	ND	0.005	ND	ND	ND	ND	ND	ND	ND	0.006
	plants	1.38	ND	1.47	ND	3.78	3.10	4.63	1.78	ND	ND	ND
Baia de Arieș upstream	water	0.004	ND	0.005	0.002	ND	ND	ND	ND	ND	ND	0.014
	plants	1.84	ND	1.01	1.18	0.76	2.48	1.82	ND	ND	ND	ND
Baia de Arieș downstream	water	0.002	ND	0.002	ND	ND	ND	ND	ND	ND	ND	0.006
	plants	13.21	4.75	1.87	2.11	3.43	ND	3.63	0.98	ND	ND	ND
Sălciua upstream	water	0.005	ND	0.008	ND	ND	ND	ND	ND	ND	ND	ND
	plants	4.05	ND	4.11	1.82	ND	ND	1.67	ND	ND	ND	ND
Sălciua downstream	water	0.003	ND	0.002	ND	ND	ND	ND	ND	ND	ND	ND
	plants	15.87	3.50	50.33	0.49	3.77	1.46	1.04	0.55	ND	ND	1.80
Câmpia Turzii upstream	water	0.024	0.005	0.005	0.0032	ND	ND	ND	ND	ND	ND	0.010
	plants	0.67	ND	0.94	0.52	0.36	1.99	1.35	ND	ND	ND	1.00
Câmpia Turzii downstream	water	0.041	0.010	0.002	0.005	0.006	0.006	0.003	ND	ND	ND	0.016
	plants	3.52	1.50	0.90	0.56	1.41	0.60	1.77	0.53	ND	ND	1.43
Turda upstream	water	0.004	ND	0.002	0.001	ND	ND	ND	ND	ND	ND	ND
	plants	1.14	ND	0.50	ND	ND	ND	0.31	ND	ND	ND	ND
Turda downstream	water	0.021	0.004	0.004	0.003	ND	ND	ND	ND	ND	ND	0.007
	plants	4.18	2.44	0.72	ND	0.97	0.71	2.06	0.20	ND	ND	0.49
Abrud flow	water	0.001	0.003	0.003	0.002	ND	ND	ND	ND	ND	ND	ND
	plants	0.82	0.97	2.24	ND	ND	ND	2.94	0.58	ND	ND	ND

DW—dry weight, ND—not detected. Limit of detection: 0.0001 μg/L.

**Table 3:** Monocyclic aromatic hydrocarbon concentrations in water (mg/L ± SD) * and aquatic plant (mg/kg DW ± SD) samples collected from the Arieş basin.

		Benzene	Toluene	Xylene	Ethylbenzene
Câmpeni upstream	water	1.58 ± 0.09	0.29 ± 0.04	ND	ND
	plants	0.59 ± 0.13	0.76 ± 0.08	ND	ND
Câmpeni downstream	water	1.12 ± 0.36	0.35 ± 0.11	0.41 ± 0.07	ND
	plants	1.48 ± 0.08	1.14 ± 0.05	ND	ND
Baia de Arieș upstream	water	2.60 ± 0.17	0.42 ± 0.08	ND	ND
	plants	2.01 ± 0.13	ND	ND	ND
Baia de Arieș downstream	water	1.84 ± 0.09	0.42 ± 0.07	0.28 ± 0.11	ND
	plants	ND	ND	ND	ND
Sălciua upstream	water	3.28 ± 0.11	0.27 ± 0.07	0.45 ± 0.05	ND
	plants	ND	ND	ND	ND
Sălciua downstream	water	0.07 ± 0.04	0.23 ± 0.08	0.42 ± 0.09	ND
	plants	ND	ND	ND	ND
Turda upstream	water	ND	0.48 ± 0.09	ND	ND
	plants	ND	ND	ND	ND
Turda downstream	water	0.75 ± 0.06	0.24 ± 0.04	ND	ND
	plants	6.98 ± 0.05	0.71 ± 0.07	3.02 ± 0.11	ND
Câmpia Turzii upstream	water	0.23 ± 0.05	0.21 ± 0.08	0.39 ± 0.07	ND
	plants	ND	ND	ND	ND
Câmpia Turzii downstream	water	ND	0.19 ± 0.05	0.50 ± 0.09	ND
	plants	ND	ND	ND	ND
Abrud inflow	water	8.01 ± 0.10	0.25 ± 0.05	0.51 ± 0.05	ND
	plants	ND	ND	ND	ND

* Data represent the mean values ± standard deviation (SD) of three replicates; DW—dry weight; ND—not detected. Limit of detection: 0.002 mg/L.

**Table 4:** Polycyclic aromatic hydrocarbon concentrations in water (μg/L ± SD) * and aquatic plant (mg/kg ± SD) samples collected from the Arieş basin.

		Nap	Acy	Flo	Ph + Ant	Pyr	BbF + BkF	BaP	BaA + Chr
Câmpeni upstream	water	ND	ND	ND	ND	ND	ND	ND	30.3 ± 0.33
	plants	ND	ND	0.34 ± 0.04	ND	ND	3.6 ± 0.32	0.81 ± 0.03	0.60 ± 0.08
Câmpeni downstream	water	ND	ND	ND	ND	ND	ND	ND	ND
	plants	ND	ND	ND	ND	0.15 ± 0.04	30.6 ± 0.16	ND	0.33 ± 0.06
Baia de Arieș upstream	water	ND	ND	ND	ND	ND	ND	ND	ND
	plants	ND	ND	ND	0.09 ± 0.02	ND	43.3 ± 0.57	ND	0.75 ± 0.05
Baia de Arieș downstream	water	ND	ND	ND	ND	ND	ND	ND	ND
	plants	ND	ND	0.30 ± 0.01	ND	ND	3.07 ± 0.05	ND	2.98 ± 0.09
Sălciua upstream	water	ND	ND	ND	2.2 ± 0.03	ND	ND	ND	ND
	plants	ND	ND	0.28 ± 0.07	ND	ND	ND	ND	ND
Sălciua downstream	water	ND	ND	ND	ND	ND	ND	ND	ND
	plants	ND	ND	0.61 ± 0.07	2.25 ± 0.08	ND	53.0 ± 3.27	ND	1.90 ± 0.24
Turda upstream	water	ND	ND	ND	ND	ND	ND	ND	ND
	plants	ND	ND	0.29 ± 0.08	ND	ND	ND	ND	ND
Turda downstream	water	ND	ND	ND	ND	ND	ND	ND	ND
	plants	ND	ND	ND	ND	ND	ND	ND	ND
Câmpia Turzii upstream	water	ND	ND	ND	ND	ND	ND	ND	ND
	plants	ND	ND	0.46 ± 0.05	ND	ND	ND	ND	0.74 ± 0.04
Câmpia Turzii downstream	water	ND	ND	ND	ND	ND	ND	ND	ND
	plants	ND	ND	ND	ND	ND	ND	ND	1.11 ± 0.06
Abrud inflow	water	ND	ND	ND	ND	ND	ND	ND	2.0 ± 0.41
	plants	ND	ND	0.47 ± 0.07	ND	ND	21.1 ± 0.04	ND	ND

* Data represent the mean values ± standard deviation (SD) of three replicates; ND—not detected; limit of detection: 0.001 mg/L; naphthalene (Nap), acenaphthylene (Acy), fluorene (Flo), phenanthrene (Ph) + anthracene (Ant), pyrene (Pyr), benzo(b)fluoranthene BbF + benzo(k)fluoranthene BkF, benzo(a)pyrene BaP, benz(a)anthracene BaA + chrysene (Chr).

**Table 5:** Trace and heavy metal concentrations in water (μg/L) and aquatic plant (mg/kg DW) * samples collected from the Arieş basin.

		Pb	Cd	Cu	Mn	Cr	Ni	Zn	Fe
Câmpeni upstream	water	ND	ND	ND	0.078	ND	ND	0.005	0.432
	plants	0.189	0.007	0.421	9.408	0.132	0.855	1.627	135.23
Câmpeni downstream	water	ND	ND	0.011	0.288	ND	ND	0.012	0.962
	plants	0.201	0.008	0.399	13.339	0.125	1.056	1.204	127.15
Baia de Arieș upstream	water	ND	ND	0.061	0.491	ND	ND	0.148	2.182
	plants	0.372	0.058	10.559	38.84	0.109	1.358	6.502	198.65
Baia de Arieș downstream	water	ND	ND	0.044	0.313	ND	ND	0.053	2.442
	plants	0.794	0.134	25.509	89.38	0.123	3.057	18.585	286.14
Sălciua upstream	water	ND	ND	0.046	0.499	ND	ND	0.07	0.962
	plants	0.419	0.053	8.037	34.034	0.167	0.856	6.432	193.26
Sălciua downstream	water	ND	ND	0.121	0.818	ND	ND	0.395	5.462
	plants	0.124	0.015	0.756	2.657	0.070	3.206	1.458	54.89
Turda upstream	water	ND	ND	0.018	0.07	ND	ND	0.015	0.259
	plants	0.366	0.004	1.230	12.016	0.209	3.317	2.314	229.52
Turda downstream	water	ND	ND	0.016	0.045	ND	ND	0.007	0.18
	plants	0.419	0.006	1.746	10.88	0.251	3.303	2.455	303.83
Câmpia Turzii upstream	water	ND	ND	0.013	0.055	ND	ND	0.009	0.122
	plants	0.527	0.007	1.895	19.458	0.365	3.283	3.427	380.43
Câmpia Turzii downstream	water	ND	ND	0.011	0.034	ND	ND	ND	0.071
	plants	0.650	0.011	2.298	25.438	0.361	2.673	4.742	384.82
Abrud inflow	water	ND	0.008	0.047	5.136	ND	ND	0.241	2.129
	plants	0.400	0.044	1.081	10.696	0.055	0.691	5.398	156.69
75/440/EEC target value; imperative value (mg/L)		0; 0.05	0.001; 0.005	0.02; 0.05				0.5; 3.0	

* DW—dry weight; ND—not detected.

**Table 6:** Growth rate of *Scendesmus opoliensis* in water (%) from the Arieș River.

Time Duration	Control	25	50	75	100	r *
Baia de Arieș downstream						
24 h	2.4	0.9	2.05	0.6	0.35	−0.72
48 h	2.95	1.1	2.8	1.55	1.1	−0.69
72 h	3.8	1.9	3.1	0.9	1.5	−0.75
Sălciua downstream						
24 h	2.4	0.85	0.45	0.4	0.9	−0.78
48 h	2.95	1.15	0.45	0.55	0.7	−0.85
72 h	3.8	1.35	0.9	0.2	1.4	−0.77
Turda upstream						
24 h	2.4	1.25	0.95	0.4	0.65	−0.89
48 h	2.95	1.3	1.15	0.5	0.65	−0.88
72 h	3.8	1.8	1.35	0.55	0.85	−0.91
Câmpia Turzii downstream						
24 h	2.4	1.25	1.15	0.75	0.2	−0.90
48 h	2.95	1.85	1.4	0.95	0.45	−0.91
72 h	3.8	2.25	1.55	1.05	0.6	−0.91
Abrud inflow						
24 h	2.4	0.7	0.3	0.1	0.4	−0.79
48 h	2.95	1.6	0.9	0.4	0.6	−0.78
72 h	3.8	2.0	1.8	0.9	1.2	−0.77

* Correlation index between the growth medium concentration and the growth rate of the algal cultures.

**Table 7:** Inhibition (%) of *Scendesmus opoliensis*-culture growth rate in water (%) based on samples from the Arieș River.

	25	50	75	100	ErC50 > *
Baia de Arieș downstream	56.67	28.21	74.80	64.73	22.05
Sălciua downstream	49.70	57.83	82.40	63.85	43.15
Turda upstream	11.91	36.17	61.70	68.08	60.70
Câmpia Turzii downstream	34.51	52.58	66.12	76.77	47.50
Abrud inflow	46.60	55.00	71.60	61.60	45.45

* The acute endpoint = concentration of chemicals that reduces the growth rate of an algal population by 50% in 72 h.

**Table 8:** The effects of water from the Arieş River on the mitotic index, frequency of mitotic divisions, chromosomal aberrations, and abnormal mitosis (% ± SD) * in onion-root meristematic cells.

	Mitotic Index	Prophase	Metaphase	Anaphase	Telophase	Chromosomal Aberrations	Abnormal Mitosis
Control	10.8 ± 1.3	37.4 ± 9.5	18.1 ± 4.8	12.2 ± 5.0	32.0 ± 7.4	0.1 ± 0.5	0.0 ± 0.0
Câmpeni upstream	10.3 ± 1.5	38.3 ± 7.0	19.6 ± 4.6	13.4 ± 3.1	27.9 ± 5.8	0.8 ± 1.4	0.0 ± 0.1
Câmpeni downstream	11.7 ± 2.4	44.3 ± 4.6	16.6 ± 3.1	11.4 ± 3.1	27.0 ± 5.2	0.6 ± 0.9	0.0 ± 0.1
Baia de Arieş upstream	10.6 ± 2.1	41.7 ± 5.7	16.3 ± 3.2	13.4 ± 4.1	27.8 ± 4.0	0.8 ± 1.1	0.0 ± 0.1
Baia de Arieş downstream	9.6 ± 1.1	44.0 ± 5.5	18.7 ± 5.3	10.6 ± 1.8	25.7 ± 7.5	0.9 ± 1.0	0.0 ± 0.1
Sălciua upstream	10.3 ± 2.4	40.9 ± 4.9	19.0 ± 3.4	10.6 ± 0.6	28.6 ± 5.3	0.9 ± 1.7	0.1 ± 0.0
Sălciua downstream	9.7 ± 1.0	40.1 ± 6.4	18.1 ± 4.7	11.7 ± 3.5	29.7 ± 5.7	0.4 ± 0.9	0.0 ± 0.0
Turda upstream	11.6 ± 1.5	44.3 ± 5.1	15.8 ± 4.1	12.2 ± 3.7	36.4 ± 3.7	1.2 ± 1.7	0.1 ± 0.1
Turda downstream	10.0 ± 1.3	45.0 ± 6.5	16.2 ± 3.6	13.2 ± 2.9	24.7 ± 5.3	0.9 ± 1.2	0.0 ± 0.1
Câmpia Turzii upstream	11.0 ± 1.3	40.3 ± 5.0	18.9 ± 3.8	12.6 ± 4.0	27.7 ± 4.7	0.4 ± 0.7	0.0 ± 0.0
Câmpia Turzii downstream	10.3 ± 1.7	37.9 ± 9.0	15.2 ± 3.7	12.9 ± 5.0	32.0 ± 9.2	1.8 ± 1.3	0.1 ± 0.1
Abrud inflow	11.2 ± 1.4	41.3 ± 4.8	18.0 ± 4.1	12.2 ± 4.3	25.9 ± 3.4	2.5 ± 1.9	0.2 ± 0.2

* Data represent mean values (%) ± standard deviation (SD).

**Table 9:** Individual carotenoid content (μg/g ± SD) in aquatic plant samples from the Arieş River.

	Total	Neoxanthin	Violaxanthin	Anteraxanthin	Lutein	Zeaxanthin	β-Carotene
Câmpeni upstream	205.50 ± 12.50	3.29 ± 0.30	0.82 ± 0.17	0.62 ± 0.09	117.14 ± 5.82	0.41 ± 0.08	84.67 ± 3.91
Câmpeni downstream	195.63 ± 12.55	10.17 ± 0.81	10.96 ± 0.63	4.11 ± 0.36	94.30 ± 4.18	1.76 ± 0.14	62.99 ± 4.23
Baia de Arieș upstream	150.50 ± 5.50	9.48 ± 0.41	6.77 ± 0.45	2.71 ± 0.18	69.08 ± 5.11	0.15 ± 0.06	61.86 ± 3.45
Baia de Arieș downstream	206.33 ± 7.51	5.57 ± 0.34	5.36 ± 0.22	2.68 ± 0.19	130.40 ± 8.30	ND	56.12 ± 3.11
Sălciua upstream	160.77 ± 10.75	10.61 ± 0.80	8.68 ± 0.56	5.14 ± 0.34	80.87 ± 4.23	0.80 ± 0.09	51.28 ± 3.54
Sălciua downstream	110.23 ± 8.75	3.42 ± 0.33	3.75 ± 0.41	0.22 ± 0.08	66.69 ± 5.14	0.44 ± 0.10	33.18 ± 1.44
Turda upstream	154.20 ± 7.52	3.70 ± 0.31	4.47 ± 0.30	3.55 ± 0.29	82.65 ± 3.87	0.46 ± 0.11	58.44 ± 2.87
Turda downstream	141.97 ± 6.01	8.38 ± 0.55	9.23 ± 0.42	2.98 ± 0.18	74.39 ± 4.25	ND	46.14 ± 1.89
Câmpia Turzii upstream	113.97 ± 8.00	3.53 ± 0.40	2.17 ± 0.33	0.91 ± 0.16	68.72 ± 5.56	0.57 ± 0.11	40.12 ± 2.18
Câmpia Turzii downstream	155.37 ± 5.04	4.51 ± 0.29	4.35 ± 0.31	0.62 ± 0.10	94.93 ± 3.66	1.24 ± 0.08	47.39 ± 2.23
Abrud inflow	210.80 ± 5.01	7.38 ± 0.48	4.43 ± 0.39	3.16 ± 0.21	108.35 ± 7.95	0.21 ± 0.07	82.00 ± 4.47

Data represent the mean values ± standard deviation (SD) of three replicates; ND—not detected.

**Table 10:** Fatty acid composition (% of total fatty acids) of phospholipids in aquatic plant samples from the Arieş River.

	Palmitic Acid C16:0	Palmitoleic Acid C16:1 (n-7)	Stearic Acid C18:0	Oleic Acid C18:1 (n-9)	Linoleic Acid C18:2 (n-6)	Linolenic Acid C18:3 (n-3)	SFA *	UFA **	DBI ***
Câmpeni upstream	37.01 ± 0.51	0.81 ± 0.07	6.73 ± 0.28	7.05 ± 0.27	16.31 ± 0.44	32.09 ± 0.52	43.74	56.26	3.13
Câmpeni downstream	42.94 ± 0.73	2.3 ± 0.11	4.26 ± 0.17	4.11 ± 0.22	17.17 ± 0.42	28.89 ± 0.24	47.20	52.47	2.70
Baia de Arieș upstream	33.27 ± 0.38	4.68 ± 0.19	15.66 ±0.50	3.75 ± 0.20	18.66 ± 0.34	23.71 ± 0.35	48.93	50.80	2.39
Baia de Arieș downstream	26.98 ± 0.30	1.21 ± 0.08	16.75 ±0.42	4.14 ± 0.26	16.02 ± 0.35	34.83 ± 0.47	43.73	56.20	3.24
Sălciua upstream	48.74 ± 0.75	2.49 ± 0.13	5.83 ± 0.15	5.93 ± 0.24	12.52 ± 0.40	24.34 ± 0.31	54.57	45.28	1.95
Sălciua downstream	28.71 ± 0.31	4.04 ± 0.25	8.85 ± 0.28	9.3 ± 0.38	20.88 ± 0.35	28.02 ± 0.35	34.54	65.26	3.71
Turda upstream	37.55 ± 0.40	3.71 ± 0.24	7.63 ± 0.24	5.65 ± 0.26	19.89 ± 0.40	25.40 ± 0.31	45.18	54.64	2.77
Turda downstream	35.14 ± 0.47	5.93 ± 0.28	17.51 ±0.49	4.77 ± 0.22	13.65 ± 0.31	22.60 ± 0.35	52.65	46.95	2.01
Câmpia Turzii upstream	30.62 ± 0.33	3.41 ± 0.11	9.12 ± 0.33	6.64 ± 0.30	21.04 ± 0.41	28.87 ± 0.36	39.74	59.96	3.49
Câmpia Turzii downstream	36.05 ± 0.35	0.28 ± 0.04	5.83 ± 0.26	2.34 ± 0.15	21.17 ± 0.33	34.24 ± 0.44	41.88	58.03	3.53
Abrud inflow	27.77 ± 0.29	0.94 ± 0.07	16.96 ±0.34	3.21 ± 0.12	15.77 ± 0.33	35.06 ± 0.41	44.73	54.98	3.15

Data represent the mean values ± standard deviation (SD) of three replicates. * SFA = Σ % SFA (Saturated Fatty Acids); ** NFA = Σ% UFA (Unsaturated Fatty Acids); *** DBI = Double Bond Index (DBI = [(1×% monoenoic FA) + (2×% dienoic FA + 3×% trienoic FA]/Σ% SFA, [39]).

**Table 11:** Fatty acid composition (% of total fatty acids) of glycolipids in aquatic plant samples from the Arieş River.

	Palmitic Acid C16:0	Palmitoleic Acid C16:1 (n-7)	Stearic Acid C18:0	Oleic Acid C18:1 (n-9)	Linoleic Acid C18:2 (n-6)	Linolenic Acid C18:3 (n-3)	SFA *	UFA **	DBI ***
Câmpeni upstream	24.76 ± 0.32	2.01 ± 0.10	11.45 ± 0.41	1.53 ± 0.10	5.41 ± 0.27	54.53 ± 1.05	36.21	63.48	4.91
Câmpeni downstream	26.01 ± 0.33	0.43 ± 0.07	1.83 ± 0.09	1.62 ± 0.11	22.05 ± 0.31	48.03 ± 1.02	27.84	72.13	6.83
Baia de Arieș upstream	27.14 ± 0.29	0.52 ± 0.06	6.04 ± 0.20	3.62 ± 0.22	12.41 ± 0.28	50.12 ± 1.12	33.18	66.67	5.40
Baia de Arieș downstream	22.85 ± 0.32	3.09 ± 0.19	8.80 ± 0.33	2.90 ± 0.14	8.00 ± 0.26	53.64 ± 1.08	31.65	67.63	5.78
Sălciua upstream	32.53 ± 0.38	0.40 ± 0.08	2.49 ± 0.17	8.77 ± 0.29	12.20 ± 0.22	43.56 ± 0.87	35.02	64.93	4.69
Sălciua downstream	29.12 ± 0.33	0.42 ± 0.06	2.29 ± 0.17	2.55 ± 0.16	16.28 ± 0.29	48.29 ± 0.75	31.41	67.54	5.74
Turda upstream	28.62 ± 0.31	0.44 ± 0.05	2.66 ± 0.14	3.24 ± 0.25	18.03 ± 0.35	46.78 ± 1.11	31.28	68.49	5.76
Turda downstream	30.49 ± 0.36	0.54 ± 0.05	2.77 ± 0.15	8.95 ± 0.28	12.4 ± 0.21	44.82 ± 1.05	33.26	66.71	5.07
Câmpia Turzii upstream	28.32 ± 0.34	0.45 ± 0.05	2.38 ± 0.18	3.75 ± 0.20	17.11 ± 0.30	47.66 ± 0.69	30.70	68.97	5.91
Câmpia Turzii downstream	20.26 ± 0.28	0.60 ± 0.08	3.88 ± 0.13	1.55 ± 0.11	7.67 ± 0.31	65.89 ± 1.21	24.14	75.71	8.91
Abrud inflow	22.95 ± 0.30	2.42 ± 0.14	12.57 ± 0.36	1.69 ± 0.09	5.12 ± 0.29	55.05 ± 0.99	35.52	64.28	5.05

Data represent the mean values ± standard deviation (SD) of three replicates. * SFA = Σ% SFA (Saturated Fatty Acids); ** NFA = Σ% UFA (Unsaturated Fatty Acids); *** DBI = Double Bond Index (DBI = [(1×% monoenoic FA) + (2×% dienoic FA + 3×% trienoic FA]/Σ% SFA), [39]).

**Table 12:** Induced chlorophyll fluorescence parameters (means ± SE) * in leaves of bean plants treated with water samples collected from the Arieș River.

	Fo	Fm	Fv/Fm	Φ
Control	254 ± 1 ^d^	1363 ± 2.68 ^f^	0.831 ± 0.014 ^a^	0.763 ± 0.005 ^a^
Câmpeni upstream	242 ± 3 ^e^	1425.6 ± 7.81 ^d^	0.828 ± 0.017 ^a^	0.765 ± 0.008 ^a^
Câmpeni downstream	276.6 ± 3.5 ^c^	1546.6 ± 4.59 ^c^	0.819 ± 0.026 ^a^	0.769 ± 0.007 ^a^
Baia de Arieș upstream	307 ± 3 ^b^	1577.6 ± 8.96 ^b^	0.814 ± 0.015 ^a^	0.755 ± 0.014 ^a^
Baia de Arieș downstream	112.4 ± 4.5 ^g^	338.6 ± 7.17 ^g^	0.654 ± 0.013 ^d^	0.623 ± 0.011 ^c^
Sălciua upstream	285 ± 4 ^c^	1417 ± 9.83 ^d^	0.789 ± 0.008 ^b^	0.750 ± 0.013 ^a^
Sălciua downstream	217 ± 4 ^f^	1379.3 ± 8.11 ^e^	0.787 ± 0.007 ^b^	0.748 ± 0.016 ^a^
Turda upstream	255.6 ± 4.5 ^d^	1484 ± 6.75 ^c^	0.817 ± 0.016 ^a^	0.757 ± 0.005 ^a^
Turda downstream	348 ± 6 ^a^	1744 ± 11.20 ^a^	0.796 ± 0.020 ^b^	0.750 ± 0.016 ^a^
Câmpia Turzii upstream	299.2 ± 4 ^b^	1632.6 ± 7.61 ^b^	0.808 ± 0.019 ^a^	0.741 ± 0.019 ^a^
Câmpia Turzii downstream	112.2 ± 6 ^g^	280.3 ± 8.06 ^h^	0.590 ± 0.009 ^e^	0.530 ± 0.021 ^d^
Abrud inflow	95 ± 4 ^h^	346.6 ± 6.28 ^g^	0.715 ± 0.012 ^c^	0.690 ± 0.017 ^b^

* Data represent mean values ± standard error (n = 5); different superscript letters indicate significant differences at the *p* < 0.05 level (separately for each column). Fo—ground chlorophyll fluorescence; Fm—transitory maximal fluorescence; Fv—variable chlorophyll fluorescence; Φ—effective quantum efficiency of PSII.

## Data Availability

Not applicable.
